# The Link Between Opioid Misuse and Suicidal Ideation Is Strongest Among Sexual Minorities

**DOI:** 10.1002/jclp.70217

**Published:** 2026-08-22

**Authors:** Katherine Musacchio Schafer, Rachel M. Schott, Julia C. Dodd, Jameson K. Hirsch

**Affiliations:** ^1^ Department of Biomedical Informatics Vanderbilt University Medical Center Nashville Tennessee USA; ^2^ Department of Psychiatry and Behavioral Sciences Vanderbilt University Medical Center Nashville Tennessee USA; ^3^ Department of Psychology East Tennessee State University Johnson City Tennessee USA; ^4^ Department of Psychiatry and Behavioral Sciences, Quillen College of Medicine East Tennessee State University Johnson City Tennessee USA

**Keywords:** LGB, opioid misuse, sexual minority people: suicidal ideation

## Abstract

Opioid misuse and suicidal ideation (SI) are significant public health concerns in the United States, and incidence rates may be elevated among sexual minority people. We used data from the 2021 National Survey on Drug and Health (heterosexual, *n* = 39,647, lesbian/gay *n* = 1329, bisexual *n* = 3774) to investigate rates of opioid misuse and suicidal ideation (SI) across sexual identity groups. We found that opioid misuse (heterosexual, 3.0%, lesbian/gay, 5.2%; bisexual, 5.7%) and SI (heterosexual, 4.0%; lesbian/gay, 13%; bisexual, 23%) were highest among bisexual participants, followed by lesbian/gay participants, followed then by heterosexual participants. Further, opioid misuse and SI were associated across sexual identity groups (entire sample: *r* = 0.08, *p* < 0.001; heterosexual: *r* = 0.07, *p* < 0.001; lesbian/gay: *r* = 0.11, *p* < 0.001; bisexual: *r* = 0.082, *p* = 0.003). Notably, the association between opioid misuse and SI was strongest among sexual minority respondents. This work confirms a deleterious linkage between opioid misuse and SI and indicates that sexual minority participants were at greater risk for opioid misuse and SI as compared to heterosexual individuals. Clinicians should be cognizant of the risk of comorbid opioid misuse and SI in their sexual minority patients, as they develop and implement intervention strategies.

## 1 Introduction

Opioid misuse is a public health crisis in America and is associated with significant morbidity and mortality. Since 2010, the number of opioid overdose deaths has risen nearly four‐fold, from approximately 21,000 to more than 80,000, in 2021 (Substance Abuse and Mental Health Services Administration, 2023). An estimated three million people in the United States meet criteria for an opioid use disorder, and drug overdose is the leading cause of accidental death in the United States, with opioids being the most common drug (Centers for Disease Control and Prevention [CDC] 2023). In addition to the opioid crisis, suicide risk, including suicide ideation (SI), suicide attempts, and death by suicide, is also a public health threat. SI precedes nearly every instance of suicide death (Van Orden et al. 2008), is associated with elevated rates of anxiety and depression (Franklin et al. 2017; Schafer et al. 2022), and is often precipitated by interpersonal, marital, and occupational dysfunction (Stack et al. 2021).

Over the last decade, research has established a robust linkage between opioid misuse and suicidal ideation. For example, in a longitudinal study of 5386 American adults, the simultaneous incidence of opioid misuse and SI increased, between 2009 and 2020 (Na et al. 2022). In a study of over 40,000 Americans, opioid misuse was significantly associated with SI (Ashrafioun et al. 2017), and SI may persist even after opioid misuse is treated (Kuramoto et al. 2012). Although such population‐based research has established a consistent association between opioid misuse and SI, fewer studies have examined this linkage at the group level, including for sexual minority groups (e.g., gay, lesbian, and bisexual), who are at higher risk for elevated rates of both opioid misuse and SI (Substance Abuse and Mental Health Survey 2023). Investigating the strength of the association between opioid misuse and SI within and between sexual minority groups would give clinicians insight into possible mechanisms that contribute to the development, persistence, or exacerbation of SI in groups that are particularly at risk. Indeed, this is particularly prevalent in LGBTQ groups in that compared to heterosexual adults, LGBTQ adults as a combined group demonstrate more than twice the prevalence of past‐year prescription opioid use disorder, with bisexual participants reporting elevated rates of opioid use disorder compared to lesbian and gay participants (Paschen‐Wolff et al. 2022). Likewise, LGBTQ adults report more than twice the prevalence of SI, again with bisexual participants reporting elevated rates compared to lesbian and gay participants (Ramchand et al. 2022).

Thus, for our study, we (1) determine the rates of opioid misuse and SI by sexual identity groups (i.e., heterosexual vs*.* gay and lesbian *vs.* bisexual respondents), (2) quantify the associations between opioid misuse and SI between sexual identity groups, and (3) compare the strength of these associations between sexual identity groups. Based on previous research, we hypothesized that (1) rates of opioid misuse and SI would be highest among sexual minority groups, (2) opioid misuse and SI would be positively and significantly associated in all sexual identity groups, and (3) the relation between misuse usage and SI will be strongest among sexual minority groups.

## 2 Methods

### 2.1 Study Design and Procedure

We investigated our hypotheses using a cross‐sectional publicly available dataset, the National Survey on Drug Use and Health (NSDUH), 2021 version, which is sponsored by the Substance Abuse and Mental Health Services Administration (SAMHSA). The NSDUH was established to assess the use of alcohol, tobacco, and illegal drugs in the non‐institutionalized American household population. The survey sample design is a stratified, multistage, area probability design. Confidential, anonymous, face‐to‐face interviews were conducted by trained interviewers, in the household, and via computerized assessment.

### 2.2 Participants

The total NSDUH 2021 dataset was comprised of 58,034 participants (male: 45.5%, *n* = 26,391; female: 54.5%, *n* = 31,643). We excluded participants who did not complete items related to sexual orientation, SI, or opioid misuse, resulting in a final sample of 44,750 participants (male: *n* = 19,844, 44.3%; female: *n* = 24,906, 55.7%). Our sample was diverse in race, ethnicity, and education level (See Table 1 for demographic frequencies). In Table 2, we report between‐ and within‐group comparisons across sexual identity groups (heterosexual, *n* = 39,647, lesbian/gay *n* = 1329, bisexual *n* = 3,774), including rates of opioid misuse within the past year (*present* = 1469; *absent* = 43,408) and SI (*present* = 2985; *absent* = 41,765).

**Table 1:** Demographic features of the sample.

Variable	*n* (%)
**Sex**	
Male	19,844 (44.3)
Female	24,906 (55.7)
**Age**	
18–20 Years Old	4622 (10.3)
21–25 Years Old	8518 (19.0)
26–34 Years Old	9006 (20.1)
35 or Older	22,604 (50.5)
**Race/Ethnicity**	
Non‐Hispanic White	28,078 (62.7)
Non‐Hispanic Black/African America	4874 (10.9)
Non‐Hispanic Native American/Alaskan Native	433 (1.0)
Non‐Hispanic Native Hawaiian/Other Pacific Islander	170 (0.4)
Non‐Hispanic Asian	2563 (5.7)
Non‐Hispanic more than one race	1727 (3.9)
Hispanic	6905 (15.4)
**Education**	
Less than high school	4100 (9.2)
High school graduate	10,439 (23.3)
Some college/Associates Degree	13,530 (30.2)
College graduate	16,681 (37.3)
**Opioid Misuse in Past Year**	
Did not misuse in the past year	43,408 (96.7)
Misused in the past year	1469 (3.3)
**Suicidal Ideation**	
Absent	41,765 (93.3)
Present	2985 (6.7)
**Sexual Identity**	
Heterosexual	39,647 (88.6)
Lesbian or Gay	1329 (3.0)
Bisexual	3774 (8.4)

**Table 2:** Demographic features of the sample by sexual identity.

Characteristic	Heterosexual (*N* = 39,751)	Gay/Lesbian (*N* = 1,334)	Bisexual (*N* = 3792)
**Race/Ethnicity**			
Non‐Hispanic White	24,870 (62.6)	836 (62.7)	2,424 (63.9)
Non‐Hispanic Black/African American	4399 (11.1)	155 (11.6)	342 (9.0)
Non‐Hispanic Native American Indian/Alaskan Native	374 (0.9)	10 (0.7)	52 (1.4)
Non‐Hispanic Native Hawaiian/Other Pacific Islander	155 (0.4)	6 (0.4)	11 (0.3)
Non‐Hispanic Asian	2392 (6.0)	45 (3.4)	132 (3.5)
Non‐Hispanic more than one race	1449 (3.6)	63 (4.7)	224 (5.9)
Hispanic	6112 (15.4)	219 (16.4)	607 (16.0)
**Opioid Misuse (Past Year)**			
Did not misuse in the past year	38,570 (97.0)	1264 (94.8)	3574 (94.3)
Misused in the past year	1181 (3.0)	70 (5.2)	218 (5.7)
**Suicidal Ideation (Past Year)**			
One or More	1978 (5.0)	188 (14.1)	909 (24.0)
None	37,773 (95.0)	1146 (85.9)	2,883 (76.0)
**Education**			
Less high school	3636 (9.1)	95 (7.1)	391 (10.3)
High school grad	9,192 (23.1)	269 (20.2)	1021 (26.9)
Some college/Associates Degree	11,801 (29.7)	443 (33.2)	1334 (35.2)
College graduate	15,122 (38.0)	527 (39.5)	1046 (27.6)
**Gender**			
Male	18,371 (46.2)	673 (50.4)	866 (22.8)
Female	21,380 (53.8)	661 (49.6)	2926 (77.2)
**Age**			
18–25 years	10,560 (26.6)	513 (38.5)	2,131 (56.2)
26–34 years	7783 (19.6)	314 (23.5)	936 (24.7)
35 years or older	21,408 (53.9)	507 (38.0)	725 (19.1)

### 2.3 Measures

#### 2.3.1 Sexual Identity

To assess sexual orientation, respondents were asked “Which one of the following do you consider yourself to be?” and could respond with the following answers: *1 = Heterosexual, that is, straight*; *2 = Lesbian or Gay*; and *3 = Bisexual*. Respondents could endorse, at most, one of the items. This single‐item assessment is consistent with previous work examining the reliability and validity of single‐item metrics of sexual identity (Wilkerson et al. 2016).

#### 2.3.2 Opioid Misuse

Respondents were classified as misusing opioids in the past year or past month if they reported using heroin or misusing prescription pain relievers in these periods. Response options for this single‐item query were *1 = Yes* and *0* = *No*. Previous work has demonstrated the reliability and validity of assessing opioid misuse with a single item (Tompkins et al. 2009).

#### 2.3.3 Suicidal Ideation

To assess SI, respondents were asked “At any time in the past 12 months did you seriously think about trying to kill yourself?” Participants’ responses were coded as *1 = Yes* and *0* = *No*. Previous research indicates that single‐item assessments can be used as reliable and valid measures of SI (Hom et al. 2016).

### 2.4 Data Analytic Plan

All analyses were conducted using SPSS 24 (IBM, 2024). First, descriptive statistics for demographic and study‐related variables were calculated. Then a Pearson correlation matrix was constructed for the entire sample and for each sexual identity group. To investigate aim 1 (i.e., to determine the rates of opioid misuse and SI by sexual identity), we evaluated proportions of participants reporting of opioid misuse and SI among sexual minority groups. Then to investigate aim 2 (i.e., to quantify the associations between opioid misuse and SI between sexual identity groups) we examined the Pearson correlation matrix. Finally, to investigate aim 3 (compare the strength of these associations between sexual identity groups) we used a two‐way ANOVA to determine if the association between opioid misuse and SI varied by sexual identity group. Given that sexual identity, SI, and opioid misuse can vary by age, sex, race, substance use disorder (SUD) treatment history, and psychological distress, we controlled for these variables in our analyses. Further, given that applying survey weights can inflate variance and reduce precision, particularly in smaller subgroups such as sexual minority populations, we opted to employ unweighted models were used to maximize statistical efficiency. Likewise, although SI was a binary variable, ANOVA was used because the outcome was coded as 0/1, allowing group means to be interpreted as proportions. Under this coding, ANOVA is equivalent to a linear probability model and tests differences in mean probabilities across groups.

## 3 Results

### 3.1 Descriptive Statistics

Descriptive statistics are reported in Tables 1 and 2. Broadly, our nationally representative sample was large, and was diverse in age, sex, race, and sexual identity, and in level of education, opioid misuse, and SI.

### 3.2 Comparing Rates of Opioid Misuse and SI by Sexual Identity

Prevalence rates of opioid misuse by sexual identity were calculated (See Figure 1). Heterosexual respondents reported the lowest rates of opioid misuse (3%), followed by lesbian and gay participants (5.2%), and followed finally by bisexual participants (5.7%). Bisexual participants were significantly more likely than heterosexual participants to report past year opioid misuse (*z* = 2.57, *p* = 0.01). All other group differences were not significant.

**Figure 1:**
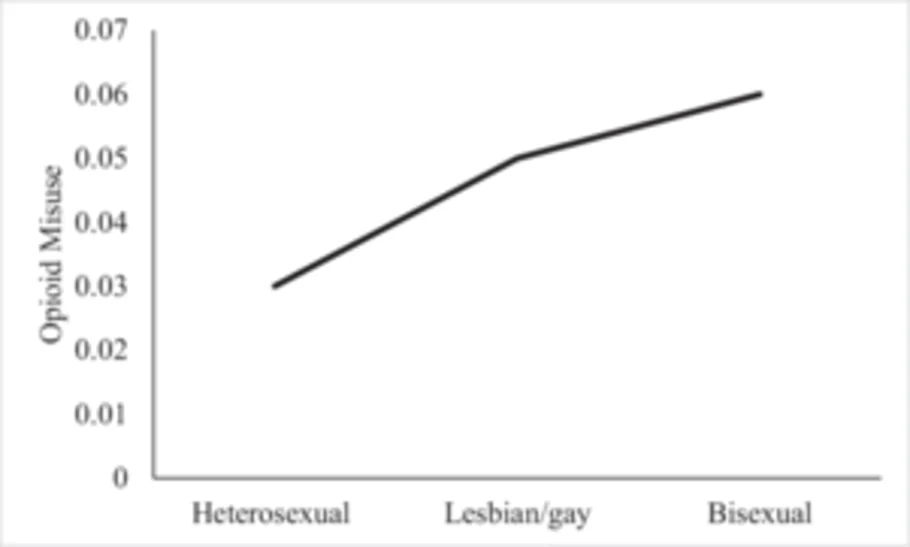
Opioid Misuse by Sexual Identity. Note. Opioid usage was measured as present or absent (i.e., ‘Have you used opioids during the past 12 months?’). Answers were coded as 1 = *yes* and 0 = *no*.

Likewise, prevalence rates of SI by sexual identity group were calculated (See Figure 2), with *Z*‐scores indicating that all group differences were statistically significant. Heterosexual participants reported the lowest rates of SI (4%), followed by lesbian and gay participants (*z* = 16.26, *p* < 0.001, 13%), with bisexual respondents reporting the highest rates (*z* = 7.93, *p* < 0.001, 23%). The difference in SI between heterosexual and bisexual participants was also significant (*z* = 48.30, *p* < 0.001).

**Figure 2:**
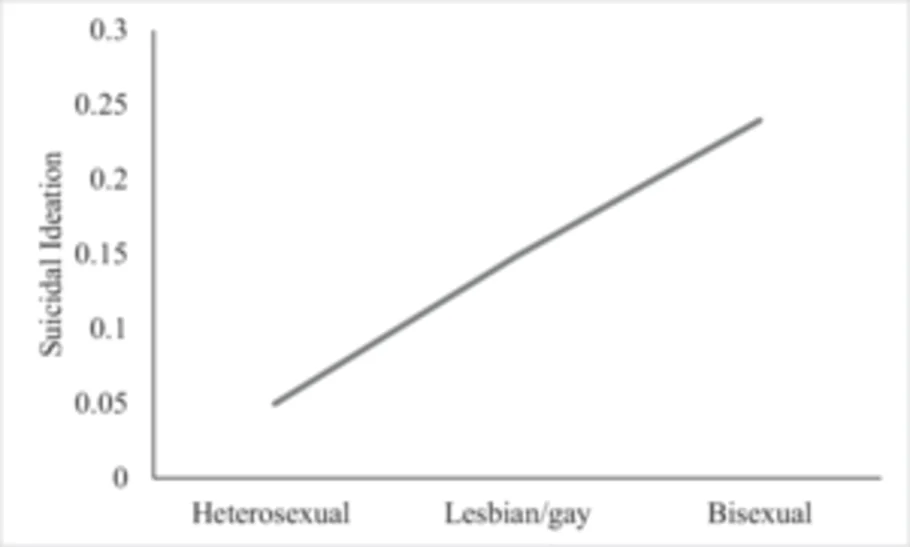
Suicidal Ideation by Sexual Identity. Note. Suicidal ideation was measured as present or absent (i.e., ‘At any time in the past 12 months did you seriously think about trying to kill yourself?’). Answers were coded as 1 = *yes* and 0 = *no*.

### 3.3 Associations Between Opioid Misuse and SI Based on Sexual Identity

Using Pearson product correlation moment analyses, the association between opioid misuse and SI was evaluated, for the entire sample as well as each sexual identity group. In the total sample, and across all sexual identity groups, opioid misuse and SI were significantly correlated (entire sample: *r* = 0.08, *p* < 0.001; heterosexual: *r* = 0.07, *p* < 0.001; lesbian/gay: *r* = 0.11, *p* < 0.001; bisexual: *r* = 0.082, *p* = 0.003).

### 3.4 SI Based on Sexual Identity, Opioid Misuse, and Sexual Identity X Opioid Misuse

Finally, a two‐way ANOVA was conducted to compare SI between sexual identities, opioid misuse (present vs absent past year use), and sexual identity x opioid misuse. The model controlled for substance use disorder treatment, psychological distress, age, gender, and race. See Table 3 and Figures 1, 2, and 3. Findings indicated that SI did not vary on the basis of sexual identity alone (*F*[6, 4486] = 14.19) or on the basis of opioid misuse alone (*F*[2, 4486] = 9.89). Instead, SI did vary on the basis of the interaction between opioid misuse and sexual identity (*F*[6, 4486] = 9.55). Notably, among these—opioid misuse, sexual identity, and their interaction—sexual identity alone had the largest effect size as it related to SI.

**Table 3:** Two‐way ANOVA of suicidal ideation by opioid usage and sexual identity.

Source	Type III sum of squares	df	Mean square	*F*	*p*
Intercept	30.17	1	30.17	8.13	0.074
SUD treatment	3.41	1	3.41	64.07	< 0.001
Psychological distress	328.96	1	328.96	6176.31	< 0.001
Gender	0.08	1	0.03	1.58	0.209
Age	11.62	1	11.62	196.1	< 0.001
Race	0.01	1	0.01	0.003	0.960
Opioid misuse	0.05	1	0.05	9.889	0.075
Sexual identity	13.39	6	6.98	14.19	0.065
Opioid misuse × Sexual identity	.998	6	0.499	9.55	< 0.001

*Note:* These are the results of the two‐way ANOVA. The two‐way ANOVA compared the strength of the relation between opioid misuse and SI, which were coded as present versus absent, by sexual identity group, which was coded as heterosexual versus gay or lesbian versus bisexual. Analyses controlled for substance use disorder treatment, psychological distress, gender, age, and race.

**Figure 3:**
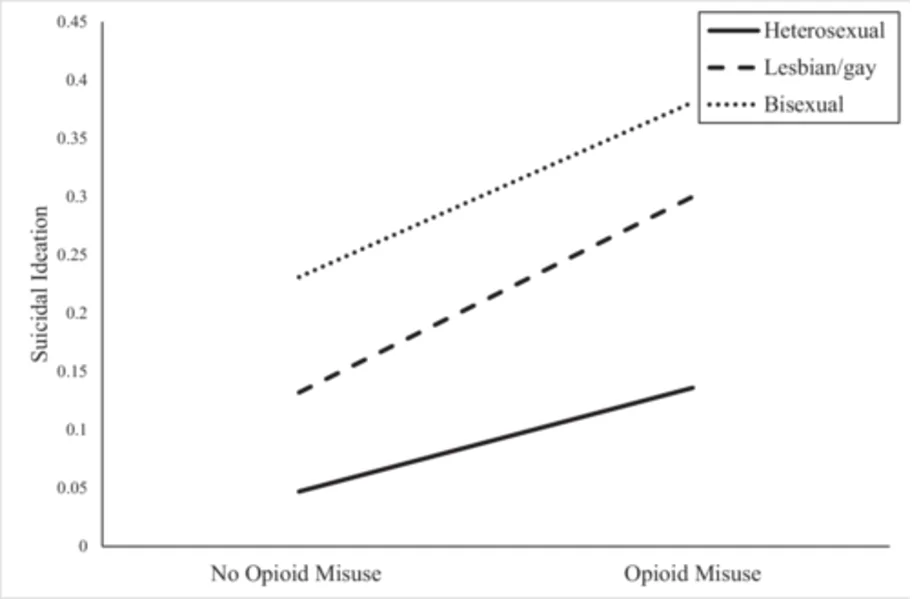
Suicidal Ideation Plotted Along the Interaction Between Sexual Identity and Opioid Usage.

Analyses indicated that age was accounted for a significant amount of the variability in SI. Sensitivity analyses were conducted such that age groups were compared (18–25 years, 26–34 years, and 35 years and older). Those findings are in Table S1. Briefly those findings again show that the interaction between opioid misuse and sexual identity was generally significant, yet sexual identity alone had the largest effect sizes among opioid misuse, sexual identity, and the interaction between the two.

## 4 Discussion

Opioid misuse and SI are public health crises in the United States, and their combined effects may be synergistically devastating. Although this deleterious association is well‐established in the larger population (e.g., Schafer et al. 2024), less research has examined this linkage as it varies across demographic groups, including sexual minority people, who have elevated rates of both opioid misuse and SI. In the current study, we (1) assessed rates of opioid misuse and SI across three sexual identity groups: heterosexual, gay and lesbian, and bisexual respondents, (2) examined the association between opioid misuse and SI across these sexual identity groups, and (3) compared the strength of the opioid‐misuse‐SI linkage between these sexual identity groups.

Findings indicated that sexual minority participants were significantly more likely than heterosexual participants to report past year opioid misuse and SI, that opioid misuse and SI were positively and significantly correlated in all sexual identity groups. However, these associations were stronger among sexual minority participants, as compared to heterosexual participants. Further, there were important differences within the sexual minority group that have bearing on implications. Bisexual respondents were significantly more likely than lesbian and gay respondents to report opioid misuse and SI, who in turn were significantly more likely to report opioid misuse and SI as compared to heterosexual respondents.

This trend indicating disparities among bisexual participants as compared to other sexual minority groups was robust and is consistent with previous literature. For example, bisexual people are at particularly elevated risk for deleterious outcomes compared to other the sexual minority demographics. Previous work (Schuler and Collins 2020) demonstrates that bisexual women have elevated rates of substance use behaviors and alcohol use disorder as compared to other sexual minority women. Bisexual people have also been shown to be at a greater risk of depression and anxiety than lesbians and gay men Ross et al. (2018). Bisexual individuals may be at particular risk for deleterious mental health outcomes due to sexual identity stress across multiple levels (e.g., intrapersonal, interpersonal, community, and societal; Chan et al. 2020).

### 4.1 Mechanisms Connecting Opioid Misuse to SI

There are a few mechanisms through which opioid misuse could associate with SI. For example, individuals who misuse opioids may experience greater frequency and intensity of psychological distress, including anxiety, depression, and loneliness, which are SI risk factors (Sampasa‐Kanyinga et al. 2021; Schafer et al. 2022). According to minority stress theory, individuals in minority groups, including sexual minority groups, experience adverse health effects resulting from oppression and discrimination, including experiences of ostracization and a lack of connectedness, which are also risk factors for substance abuse and suicide (Kaniuka et al. 2019). The Interpersonal Theory of Suicide (IPTS; Van Orden et al. 2008, 2010) supports this assertion, proposing that perceived burdensomeness and thwarted belongingness, or the lack of reciprocally supportive relationships, are robust indicators of SI, including in sexual minority samples (Fulginiti et al. 2020).

Further, IPTS asserts that acquired capability is a requisite factor for suicide attempts (Ribeiro and Witte 2014; Smith and Cukrowicz 2010), and substance abuse, including opioid misuse, is posited to contribute to development of acquired capability for suicide (Anestis et al. 2019). Our findings, therefore, can be contextualized within the frameworks of these sociocultural and interpersonal theories of stress, substance abuse, and suicide risk, with critical implications for future research and intervention efforts. Further, the bidirectional association between opioid use and SI may be predicated on psychological distress, suggesting that clinicians should routinely assess for substance abuse and suicide risk in patients experiencing psychopathology.

Given that members of sexual minority groups are at increased risk for opioid misuse and SI, we recommend that clinicians routinely screen sexual minority patients for these clinical symptoms and employ identity‐affirming interventions to prevent substance abuse and death by suicide in the LGBTQ population (McGeough 2021; Mustanski and Espelage 2020).

### 4.2 Clinical Implications

Clinical assessment of opioid misuse and SI should be implemented with greater consideration of an individual's social identity and their existence within a broader ecological system. Bronfenbrenner (1979) proposed ecological systems theory to explain how an individual's development and behavior is shaped by interactions between their identity and environmental systems. Indeed, minority stress exists across all levels of an individual's ecosystem; therefore, understanding the context in which an individual exists and navigates their environment may provide a deeper understanding of their opioid misuse and SI. Xin et al. 2023) reviewed the problematic substance use literature and found that minority stress was associated with the use of substances as a coping strategy.

Indeed, SI has been described by many as an intensely distressing experience, and groups who experience elevated rates and intensity of SI (including sexual minority people), may be more likely to engage in opioid misuse to soothe those feelings of distress. A recent study (Resnik et al. 2024) that employed computation linguistics to understand reasons why people lived, even when they were at their most suicidal points to this mechanism of action linking opioid misuse to SI. Using a dataset with responses from over 16,000 Reddit users who experienced severe SI, but lived through that intense distress, researchers found that self‐soothing behaviors including but not limited to substance use—helped people endure their darkest and most suicidal moments. “Getting high,” “feeling euphoric,” and even “looking forward to getting high” were specifically listed as reasons why people were able to endure their most intense suicidal experiences. Understanding the function of substance use and how it may relate to SI helps tailor treatment outcomes; however, if clinical conversations occur without consideration or assessment of minority stress and environmental contexts, effective individualized treatment becomes less accessible.

Identity‐affirming approaches to treatment may reduce barriers to care and increase patient satisfaction. According to a qualitative analysis of LGBTQ+ experiences in substance use services, participants described various barriers to efficient and affirming care, including name‐calling, sexual harassment, misgendering, and more (Paschen‐Wolff et al. 2024). Notably, some participants reported that these experiences of minority stress led to their return to use, demonstrating how providers can inadvertently exacerbate substance use and risk. While most participants described obstacles to navigating substance use treatment with a queer identity, several individuals described how access to LGBTQ + ‐affirming social support increased their comfort and feelings of acceptance (Paschen‐Wolff et al. 2024). While opioid misuse and suicide risk affect all populations, it is imperative for clinicians to recognize the bidirectional relationship between opioid misuse and SI, particularly among sexual minorities, given their heightened vulnerability associated with identity‐related stressors. Although more research is needed to identify effective intervention strategies tailored to LGBTQ+ individuals, current treatment approaches should employ an identity‐affirming framework that recognizes contextual factors in order to avoid exacerbating harm and further increasing risk for these behaviors.

## 5 Limitations

Despite novel findings, our work must be considered within the context of limitations. Although we utilized a large, diverse dataset and well‐powered analyses, our assessment of SI, opioid misuse, and sexual identity via single item self‐report measures is less than ideal. Previous psychometric investigation indicates that these constructs can be reliability and validly assessed via a single item; however, such an approach may fail to capture nuanced facets of sexual identity, substance use, and suicidality and, thus, in future work, we recommend utilization of comprehensive multi‐item measures. Our study was also cross‐sectional, which limits ability to infer temporal precedence of study variables; however, extant longitudinal research in nationally representative samples indicates that SI contributes to opioid risk behavior (Cleland et al. 2020) and, similarly, opioid use, including prescription opioid use, is predictive of suicide risk, over time (Santaella‐Tenorio et al. 2022). Prospective, longitudinal research assessing opioid use and suicide‐related symptoms in sexual minority groups, is necessary to substantiate our findings.

Our use of a diverse, nationally representative sample is a strength of our methodology, but it should be noted that this data was collected in 2021, during the COVID‐19 pandemic, which was marked by elevated rates of psychopathology, including opioid misuse and SI (Pathirathna et al. 2022; Schafer et al. 2022; Slavova et al. 2020), and such data may not be reflective of a nation that is recovering from a pandemic.

Finally, we utilized an adult dataset, yet children and adolescents, including sexual minority youth, are not immune from the burden of the opioid crisis and suicide risk. Indeed, suicide is the second leading cause of death among adolescents, who have the fastest growing rate of suicide across all age groups (CDC, 2023). Adolescents also comprise the second largest group of opioid deaths over the last 5 years (CDC, 2023). Such patterns suggest that researchers should, in future work, expand investigation of the opioid‐suicide linkage in this age group.

In closing, in a large, diverse, nationally representative sample, we found that opioid misuse and SI were associated across all sexual identity groups, but that this linkage was stronger for sexual minority respondents, particularly bisexual individuals. Our findings suggest a profile of risk among sexual minority individuals who may experience concurrent opioid risk behaviors and SI and who, as a vulnerable population, may require a multifaceted and inclusive approach to assessment and treatment that integrates an understanding of how social identity intersects mechanistically with opioid misuse and suicide risk.

## 6 Conclusion

This project employed a large, diverse nationally representative sample, the National Survey on Drug Use and Health. Within this sample, sexual minority participants endorsed elevated rates of opioid misuse and SI compared to heterosexual participants. Further, while opioid misuse and SI were positively and significantly correlated among every sexual identity group, this relationship was stronger among sexual minority participants as compared to heterosexual participants. Together our findings indicated that people who identified as gay, lesbian, or bisexual who engaged in opioid misuse endorsed elevated experiences of SI. Although these findings do not assess the temporal precedence between these constructs they may hint at potential mechanisms towards the development of SI in sexual minority adults. Further, our findings have relevance to the potential bidirectional relationship between opioid misuse and SI in sexual minorities, with the possibility that opioid misuse and SI may contribute to each other over time in a self‐amplifying cycle.

## Ethics Statement

With regards to ethics, all data are de‐identified and privacy is paramount. Full names are never recorded and answers to the survey questions are encrypted and never linked to actual addresses. Each participant's responses are combined with the responses of thousands of other people and reported only as overall numbers. Given the de‐identifying nature, the project was found to be exempt from IRB approval.

## Supporting information


**Table S1:** Two‐way ANOVA of Suicidal Ideation by Opioid Usage and Sexual Identity by Age Group.

## Data Availability

The data that support the findings of this study are openly available in the National Survey on Drug Use and Health (NSDUH) website at https://www.samhsa.gov/data/data-we-collect/nsduh-national-survey-drug-use-and-health/datafiles.
