# Supplementary material for: The Link Between Opioid Misuse and Suicidal Ideation Is Strongest Among Sexual Minorities
Source: J Clin Psychol. 2026 Aug 22;82(12):2040–7. doi: 10.1002/jclp.70217 (PMC13733649; doi:10.1002/jclp.70217)
Supplement: Supplementary file 1 [file JCLP-82-2040-s001.docx]

Supplementary Table 1.

*Two-way ANOVA of Suicidal Ideation by Opioid Usage and Sexual Identity by Age Group*

| **Source** | **18–25 Years** | **26–34 Years** | **35 Years and Older** |
| --- | --- | --- | --- |
|  | F(df1, df2), p | F(df1, df2), p | F(df1, df2), p |
| Intercept | 5.40 (1, 5.26), .065 | 1.48 (1, 2.00), .348 | 4419.12 (1, 7.71), < .001 |
| SUD Treatment | 40.60 (1, 13961), < .001 | 2.96 (1, 9571), .085 | 15.88 (1, 23707), < .001 |
| Psychological Distress | 2191.50 (1, 13961), < .001 | 1157.66 (1, 9571), < .001 | 2196.98 (1, 23707), < .001 |
| Sex | 0.08 (1, 13961), .777 | 1.33 (1, 9571), .250 | 0.37 (1, 23707), .544 |
| Race | 0.56 (1, 13961), .456 | 0.12 (1, 9571), .727 | 1.72 (1, 23707), .190 |
| Past Year Opioid Misuse | 0.01 (1, 121.33), .938 | 4.81 (1, 12.94), .047 | 2.70 (1, 20.04), .116 |
| Sexual Identity | 8.44 (6, 6.08), .010 | 2.71 (6, 5.41), .137 | 4.89 (6, 5.95), .038 |
| Opioid Misuse * Sexual Identity | 2.06 (6, 13961), .054 | 5.26 (5, 9571), < .001 | 2.37 (5, 23707), .037 |
